# Towards increased visibility of multimorbidity research

**DOI:** 10.15256/joc.2016.6.80

**Published:** 2016-03-24

**Authors:** Aline Ramond-Roquin, Martin Fortin

**Affiliations:** ^1^Department of Family Medicine and Emergency Medicine, Université de Sherbrooke, Sherbrooke, Quebec, Canada; ^2^Centre intégré universitaire de santé et de services sociaux du Saguenay–Lac-Saint-Jean, Chicoutimi, Canada; ^3^Department of General Practice, University of Angers, L’Université Nantes Angers Le Mans, Angers, France; ^4^Laboratory of Ergonomics and Epidemiology in Occupational Health, University of Angers, L’Université Nantes Angers Le Mans, Angers, France

**Keywords:** multimorbidity, comorbidity, chronic disease, bibliometrics, indexing

## Growing interest for multimorbidity

The number of people living with comorbidity, multimorbidity, or multiple chronic conditions, hereafter referred to as “multimorbidity” (see Box 1) [1, 2], has become the norm rather than the exception in healthcare. In developed countries, approximately one in four adults have at least two chronic conditions [3, 4], and over half of older adults have three or more [5]. Although the prevalence of multimorbidity increases with age, many studies have reported high rates of multimorbidity even among younger adults [6].

**Box 1. Different terms used to refer to multiple chronic conditions.**

**Multimorbidity** refers to the coexistence of multiple chronic conditions in a given individual. In its strict sense, **comorbidity** refers to additional condition(s) in an individual who has a given index disease, but is also sometimes used to refer to the concept of multimorbidity [1, 2].

Multimorbidity negatively impacts patient outcomes, including physical and psychological functioning, quality of life, and life expectancy [7, 8]. It also complicates treatment and increases healthcare utilization and costs [9–11]. Despite representing a large – and growing – proportion of adults seen in primary care today, there is a major gap in our understanding of how best to address, meet, and satisfy the complex care needs of patients with multimorbidity [11]. The traditional single-disease model of care does not work for them, and multimorbidity should definitively not be considered as the simple juxtaposition of independent conditions [12, 13].

Fortunately though, interest in multimorbidity is growing worldwide, and has become a healthcare and research priority [14, 15]. An international community interested in multimorbidity research has recently emerged and become organized through different activities, such as the creation of the *Journal of Comorbidity*, a weblog that hosts and supports the exchanges from the International Research Community on Multimorbidity [16], the organization of an international forum [17] at the North American Primary Care Research Group (NAPCRG) congress, and the publication of an “ABC of Multimorbidity” [1]. 

## Multimorbidity in the literature

**Figure 1:**
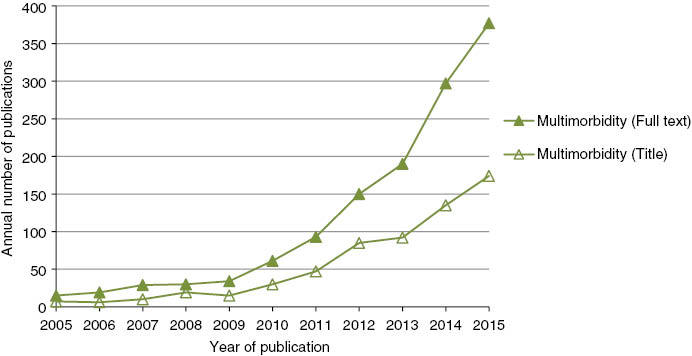
Number of publications indexed in MEDLINE between 2005 and 2015, which include the term “multimorbidity” in their full text or in their title.

At the same time, the volume of scientific literature on multimorbidity has exploded during the last decade. The number of publications using the term “multimorbidity” in MEDLINE^®^ – that were still extremely marginal some years ago – has recently shown an exceptional increase (Figure 1). Between 2009 and 2015, it has increased by a factor of 11 (in comparison with an increase by a factor 1.5 for the total number of publications in MEDLINE during the same period).

**Figure 2:**
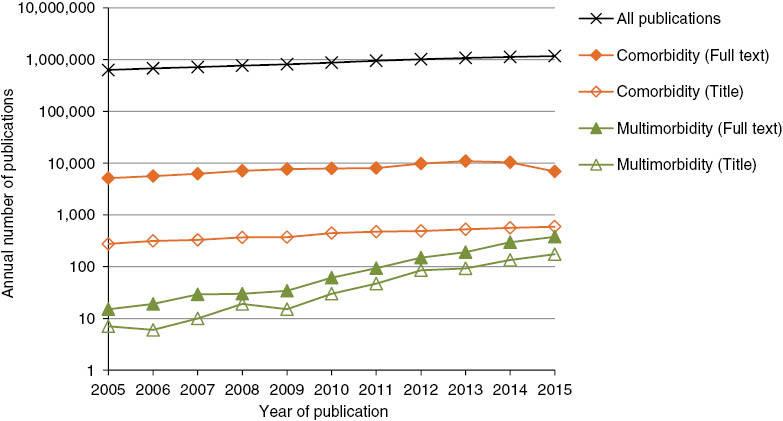
Number of publications indexed in MEDLINE between 2005 and 2015, which include the terms “comorbidity” or “multimorbidity” in their full text or in their title. *Unlike Figure 1, results are presented on a **decimal logarithmic scale**, meaning that each one-line increase is associated with a multiplication by a factor of 10. Therefore the line slopes represent relative (rather than absolute) increases over time.* The total number of publications indexed in MEDLINE is provided as a reference for the general increase of scientific production over time.

As the term “comorbidity” is sometimes used to refer to the concept of multimorbidity [2], Figure 2 presents the number of publications indexed in MEDLINE between 2005 and 2015 which include the term “multimorbidity” in comparison with those including the term “comorbidity”. Several observations can be made from this comparison. First, contrasting with the previous results, the number of publications using the term “comorbidity” has “only” increased with similar proportions as the total number of publications indexed in MEDLINE, namely between 5 and 10% each year. Secondly, the number of publications using “comorbidity” still largely exceeds that of publications using “multimorbidity”, but the gap has continuously and significantly lessened.

Finally, the number of publications with a title that includes the term “multimorbidity” represents about 50% of publications that include the same term in the full text, while this percentage is much lower, namely about 5%, for the term “comorbidity”. This suggests that the term “multimorbidity” may be more specific of research primarily focusing on multiple chronic conditions, in comparison with “comorbidity”. This hypothesis is supported by a previous bibliometric analysis focusing on publications indexed in MEDLINE during the period 1970–2012 [2]. Among 67,557 publications using the term “comorbidity”, only 1,028 (1.5%) were indexed with comorbidity as a “Major Descriptor” in the National Library of Medicine’s vocabulary thesaurus, “Medical Subject Headings (MeSH)”. In addition, this latter analysis revealed that 17% of the publications indexed with comorbidity as a “MeSH Major Descriptor” did not refer to an index disease under study and thus did not use the term in its strict sense.

## Coexistence of terms for multimorbidity

Previous bibliometric results demonstrate that the growing interest for multimorbidity has actually translated into more research in the field. However, it also raises an issue in relation to the coexistence of different terms. On the one side, the term “multimorbidity”, although not yet being very common in the literature, has recently shown a significant increase, and seems to be quite specific to the field. On the other side, the term “comorbidity” is associated with a traditionally high volume of scientific literature, but may lack specificity. In addition, even when it is used to report research primarily focusing on multiple chronic conditions, it has been shown to sometimes refer to the concept of comorbidity and other times to that of multimorbidity [2].

This situation is not surprising, since concern for multiple chronic conditions first emerged in a context where the healthcare system (and health research) was based on a disease-centered model. This has naturally led to the traditional “comorbidity” approach. More recently, there has been a strong movement towards more person-centeredness in healthcare as well as in research, in which the “multimorbidity” approach is more aligned [18]. This movement has led to a semantic transition, which has just begun, as demonstrated by the bibliometric trends. 

This coexistence of terms generates some confusion, which we believe may be prejudicial to the visibility of multimorbidity research, and eventually to its impact on healthcare and on patients. In accordance with other authors in the field, we call for preferentially using the term “multimorbidity” each time it is relevant and for limiting the use of “comorbidity” to its strict sense [19]. In this regard, the creation of a MeSH “multimorbidity” for searching articles in MEDLINE would be an inestimable support. Similarly, one may question the name of the *Journal of Comorbidity*, whose real content might be more accurately represented by the term “multimorbidity”. 

## A unique journal for multimorbidity

Independently of its name, the *Journal of Comorbidity* is of unequalled interest for all of those, like us, who have a particular interest in multimorbidity and are actively involved in its research. This is the only journal that specifically addresses multimorbidity. In that sense, it uniquely allows any stakeholders interested in the field to keep abreast of the latest developments and provides a primary source for the dissemination of research findings. 

The *Journal of Comorbidity* is currently indexed in many searchable databases and directories, but is not yet in the MEDLINE database. Increasing the volume of its published content may substantially contribute to compliance with the requirements outlined by MEDLINE. As indexing in MEDLINE is key to helping any journal seeking the widest dissemination of its content, we encourage all researchers, clinicians, and other stakeholders with an interest in multimorbidity to consider the *Journal of Comorbidity* for publishing their research. 

The existence of an authoritative resource for multimorbidity publications would constitute an inestimable lever to increase the visibility and the impact of research on multimorbidity, with the potential to eventually improve the lives of those with multimorbidity.
